# Classification of EEG Signals Reveals a Focal Aftereffect of 10 Hz Motor Cortex Transcranial Alternating Current Stimulation

**DOI:** 10.1093/texcom/tgab067

**Published:** 2022-01-07

**Authors:** Elinor Tzvi, Jalal Alizadeh, Christine Schubert, Joseph Classen

**Keywords:** classification, EEG, machine learning, motor network, transcranial alternating current stimulation

## Abstract

Transcranial alternating current stimulation (tACS) modulates oscillations in a frequency- and location-specific manner and affects cognitive and motor functions. This effect appears during stimulation as well as “offline,” following stimulation, presumably reflecting neuroplasticity. Whether tACS produces long-lasting aftereffects that are physiologically meaningful, is still of current debate. Thus, for tACS to serve as a reliable method for modulating activity within neural networks, it is important to first establish whether “offline” aftereffects are robust and reliable. In this study, we employed a novel machine-learning approach to detect signatures of neuroplasticity following 10-Hz tACS to two critical nodes of the motor network: left motor cortex (lMC) and right cerebellum (rCB). To this end, we trained a classifier to distinguish between signals following lMC-tACS, rCB-tACS, and sham. Our results demonstrate better classification of electroencephalography (EEG) signals in both theta (θ, 4–8 Hz) and alpha (α, 8–13 Hz) frequency bands to lMC-tACS compared with rCB-tACS/sham, at lMC-tACS stimulation location. Source reconstruction allocated these effects to premotor cortex. Stronger correlation between classification accuracies in θ and α in lMC-tACS suggested an association between θ and α efffects. Together these results suggest that EEG signals over premotor cortex contains unique signatures of neuroplasticity following 10-Hz motor cortex tACS.

## Introduction

Transcranial alternating current stimulation (tACS) has gained interest in the neuroscientific community as a tool to modulate endogenous oscillations in a frequency-specific manner and a range of behaviors (Tavakoli and Yun 2017). TACS affects endogenous oscillations “online,” during the stimulation, and “offline,” referring to effects that persist or arise after the stimulation has been terminated. It has been hypothesized that “online” effects occur through entrainment, that is, alignment of brain oscillations to the periodic external signal by tACS. In contrast, “offline” effects occurring at the target location are likely to be due to neuroplasticity, possibly through spike-timing dependent plasticity. Entrainment has been previously explored during simultaneous tACS-electroencephalography (EEG) experiments and has been demonstrated in animals LFPs (Krause et al. 2019; Johnson et al. 2020) as well as in humans (Helfrich et al. 2014). Although most tACS-EEG studies examine “online” effects, it is important to note that entrainment is difficult to assess since the EEG signal is strongly distorted by the stimulation artifact, and this artifact is not easily removed (Noury et al. 2016). “Offline” effects of tACS on the other hand, are more easily detected, because of the absence of stimulation artifacts. Despite the importance of “offline” aftereffects as a potential marker for neuroplasticity following tACS, only limited evidence exists regarding its focality, state-dependence, and network properties. Aftereffects of tACS ought to be well studied and clearly defined as an important step towards the use of tACS in therapeutic settings.

Previous experiments investigated “offline” aftereffect by applying tACS at 10 Hz or the individual α frequency (iAF) over parieto-occipito areas during rest (Zaehle et al. 2010; Kasten et al. 2016), as well as during task performance (Neuling et al. 2012; Helfrich et al. 2014; Vossen et al. 2015; Berger et al. 2018). These studies found posterior α power increase in post- compared with pre-stimulation blocks, during either rest or task performance. However, over the sensorimotor cortex, an opposite effect was demonstrated: iAF-tACS applied over centro-parietal areas during rest led to a decrease in μ power, that is, 8–12 Hz oscillatory power over the motor cortex (Gundlach et al. 2017). Similarly, Marshall et al. (2011) showed that 5-Hz tACS over the frontal cortex during non-rapid eye movement sleep led to a wide-spread decrease in slow (0.5–4 Hz) oscillatory power as well as decreased alpha power, specifically around stimulation location. Moliadze et al. (2019) demonstrated a location-specific aftereffect of prefrontal 10-Hz tACS during a phonological task as well as resting-state specifically in the theta (4–7 Hz) band. Thus, it seems that tACS induces topographically heterogeneous aftereffects that depend on the stimulation site and may induce modulations of oscillatory activity in other frequency bands. What is the mechanism that underlies modulation of oscillatory activity in frequencies beyond the actual stimulation frequency? Helfrich et al. (2016) found that “online” alpha tACS increased region-specific alpha-gamma phase amplitude coupling, suggesting that cross-frequency coupling may also drive “offline” aftereffects of tACS.

In this study, we aimed to address the above open questions by investigating aftereffects on the motor network induced by 10-Hz tACS of left motor cortex (lMC) in a previously published data-set (Schubert et al. 2021). Results were compared with an active (tACS to right cerebellum) and an inactive (“sham” stimulation) control condition. The cerebellum plays a fundamental role in movement control (Bastian 2006), and its connection with the motor cortex via the dentato–thalamo–cortical tract has been thoroughly studied in both animal (Kelly and Strick 2003) and human models (Tzvi et al. 2014) as well as using noninvasive stimulation approaches (Ugawa et al. 1991). The advantage of including the cerebellum as an active stimulation site is the ability to draw more specific conclusions regarding the localization of “offline” aftereffects. Subjects were stimulated while performing a serial reaction time task (SRTT), entailing both sequence and non-sequence blocks, shown to induce activity within motor cortex-cerebellar loops (Tzvi et al. 2014; Schubert et al. 2021). Specifically, we showed that this task induces changes in alpha power (Tzvi et al. 2016; Tzvi et al. 2018; Schubert et al. 2021) that may be more readily modulated by externally applied 10-Hz tACS. The effect of tACS on “offline” EEG signals was studied during resting-state as well as during performance of a non-sequence block of the SRTT.

To this end, we employed multivariate pattern analysis (MVPA), a method that extends beyond standard univariate techniques by exploiting the interactions between multiple features of the EEG signal, such as spectral profiles across multiple channels or sources, using machine-learning algorithms (Bzdok et al. 2018). MVPA has an advantage over classic parameter estimation in that it allows data-driven analyses with few a-priori hypotheses regarding spatial or temporal patterns. Here, we tested whether a classifier can discriminate short epochs of oscillatory activity recorded during task performance or resting-state, after tACS has terminated, between specific tACS protocols. Thus, it provides a means to statistically assess differences in oscillatory patterns between stimulation protocols. We hypothesized that tACS-aftereffects are: 1) most unequivocally detected at or near the location of the stimulation, 2) specific to the stimulation frequency, and 3) evident during a visuomotor task as well as rest. In addition, we investigated whether effects found in frequency bands beyond the stimulation frequency are driven by cross-frequency coupling effects as previously suggested by Veniero et al. (2015).

## Methods

### Participants and Experimental Design

**Figure 1:**
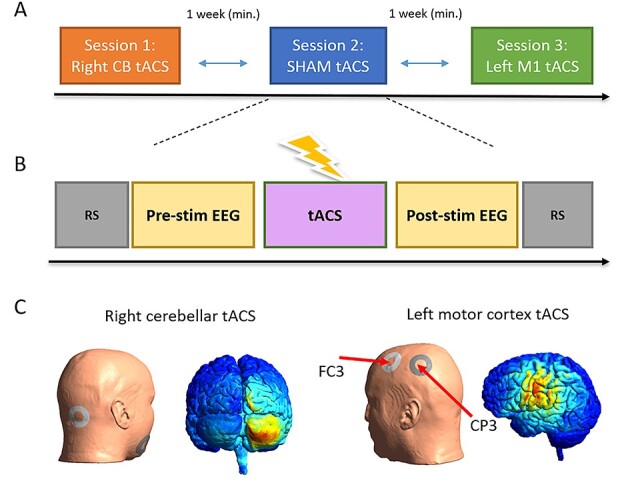
Experimental design. (*A*) Experimental sessions were kept at least 1-week apart and were counter-balanced between subjects. (*B*) Each session was divided into a pre-stimulation and post-stimulation segments, each with a resting-state recordings (both eyes-closed and eyes-open, marked in gray) as well as the SRTT (marked in yellow). (*C*) Computational modeling of electric field distribution in left MC and rCB during tACS.

The study was approved by the Ethics Committee of Leipzig University and was performed in accordance with the Declaration of Helsinki. Written informed consent was obtained prior to study participation. Further details on participant inclusion and experimental design can be found in (Schubert et al. 2021). In short, 25 healthy participants (age range: 18–38, mean age: 24.8 years, nine males) received 10-Hz tACS to either lMC or right cerebellum (rCB) as well as sham stimulation in alternate to either location (Fig. 1), in separate experimental sessions at least 1-week apart. One participant was excluded due to a technical error in the data acquisition resulting in a total sample of 24 participants.

### Serial Reaction Time Task

During tACS and at EEG recordings prior and following tACS, participants performed a modified version of the SRTT. In each trial, four squares were presented on a gray background in a horizontal array, with each square (from left to right) associated with one of four fingers of the right hand (see Supplementary Fig. 1*A*). At stimulus onset, one of the squares turned blue and the rest remained black. Participants were instructed to respond to this blue colored square with the corresponding button, as precisely and quickly as possible. The stimulus remained on the screen until a button press was registered. In case of a wrong button press, the blue colored square turned to red to mark the error. The response–stimulus interval was 500 ms (see Supplementary Fig. 1*B*). Trials were counted as correct when the appropriate key was pressed within 1000 ms after stimulus onset. In case no button was pressed within this time frame, a text appeared on the screen requesting the participants to be faster (“Schneller!”).

The task consisted of three different conditions: simple (SMP), random (RND), and sequence (SEQ). In SMP, stimuli were presented in a simple order of button presses 4–3–2–1–4–3–2–1. In RND, stimuli were presented in a pseudorandom order, generated using Matlab (the Mathworks®, Natick, MA), such that items appeared exactly twice, were not repeated and pairs of consecutive stimuli were followed by some other stimuli, thereby preventing learning by pairwise associations (Curran 1997). In SEQ, stimuli were organized in an 8-items-sequence (4–1–4–2–3–1–3–2) also preventing pairwise associations.

During tACS, participants performed a total of eight blocks of SEQ with 120 trials each. In between the SEQ blocks, three RND blocks with 40 trials each were inserted as a behavioral marker. At stimulation onset, a SMP block (40 trials) was performed. The specific order of the blocks can be observed in Supplementary Figure 1*C*. During EEG recordings prior to tACS, participants performed two RND blocks with 80 trials each, and following tACS two RND (80 trials) and two SEQ (120 trials) blocks in alternating order. For the current analyses only the second RND block PRE-tACS and the first RND block POST-tACS were analyzed.

Before and after task performance, 64-channel EEG was collected during resting-state with eyes-open (RSEO) or eyes-closed (RSEC), each with a duration of 200 s.

### tACS Protocol

tACS was applied (DC-Stimulator PLUS, NeuroConn) via two ring-shaped conductive rubber electrodes with an outer diameter of 48 mm, and an inner diameter of 24 mm (area: 15 cm^2^) and an intensity of 1 mA at 10 Hz (peak-to-peak-amplitude; sinusoidal waveform; and 0.07 mA/cm^2^ current density) for a total duration of 20 min. For rCB-tACS, one electrode was placed on the right mandibula and the other 1-cm below and 3-cm right to the inion. For lMC-tACS, one ring-shaped electrode was placed around electrode FC3 and one around CP3 rendering the current flow as precisely as possible to C3 (Fig. 1*C*) as shown by computational simulations (see details in Schubert et al. 2021, and Supplementary Materials, Section 2). The EEG cap was fitted on top of the stimulation electrodes such that recording electrodes FC3 and CP3 were placed precisely in the middle of each ring stimulation electrode. For sham stimulation, the current was ramped up for 30 s, then stayed at 1 mA for 10 s, and ramped down for another 30 s, in order to effectively blind the participants to the experiment protocol. Adverse effects by stimulation and blindness to the stimulation protocol was evaluated using questionnaires (see details in Schubert et al. 2021, and see Supplementary Materials, Section 3).

### E‌EG Recordings and Preprocessing

EEG was recorded using Ag/AgCl electrodes embedded in a 64-channel cap and connected to an eegoTM amplifier (ANT Neuro b.v.) with a sampling rate of 512 Hz and 24bit resolution. Electrodes were placed according to an extension of the international 10–20 system, and thus there was no coverage of cerebellar locations. A low-pass filter was applied at 0.26*sampling rate (*f*_cutoff_ ≈ 133 Hz). Eye movements were recorded with an electrooculogram below the left eye. EEG was recorded against an online reference electrode in location CPz. Preprocessing and all subsequent analyses were performed using in-house MATLAB® (The MathWorks) scripts and the EEGLAB toolbox (Delorme and Makeig 2004). Signals were band-pass filtered (*F*_cutoff_ = 1–49 Hz) to remove slow drifts and power line noise and re-referenced offline to the average of the signal from left and right mastoids. The signal from electrode CPz was re-calculated. Next the signals were segmented into 3-s epochs for the task-based data, −1–2 s around stimulus onset (see Supplementary Materials, Section 1, for more details), and 4 s (nonoverlapping) epochs for the RSEO and RSEC data. Using ICA, 3–4 components related to eye blink artifacts were identified and removed. Additional artifacts were removed using a simple threshold (−70 μV, +70 μV) on the filtered data and finally, the signals were re-referenced to a common-average reference.

### EEG Spectral Power and MVPA

**Figure 2:**
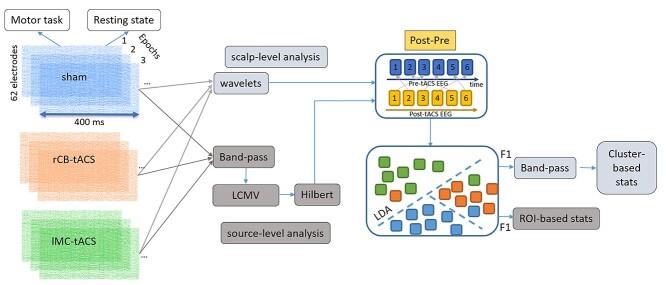
Analysis pipeline. Short EEG epochs in each stimulation protocol were analyzed on both scalp level (using electrodes, light gray) and source-level (using reconstructed voxels, dark gray). Post–pre power differences were created by shuffling the order of epochs along the time axis for PRE and POST. Data points were then analyzed using a multi-class LDA, which produced a classification accuracy (*F*1-score) for each stimulation protocol. Differences in accuracies were analyzed on scalp-level using cluster-based statistics, and on source-level using a ROI-based approach.

We used the Fieldtrip integrated MVPA-light toolbox (Treder 2020) to perform MVPA, using regularized multiclass linear discriminant analysis (LDA) classifier. Note that different classifiers, such as support vector machine (SVM), may produce better classification results that would allow a stronger differentiation between the three classes. We opted for LDA as this algorithm provides a relatively straightforward approach for multiclass classification when compared with SVM. Nonetheless, we provide in the Supplementary Materials, Section 2, a comparison of performance by both algorithms when accounting for two classes only (namely, MC tACS and sham). In Figure 2, we provide an overview of the analysis pipeline for both scalp and source-based signals. First, we computed the power spectrum of EEG signals, in each trial, in a 400-ms time window from stimulus onset, using a Morlet wavelet. These EEG segments are referred to as epochs. Signals were filtered to obtain oscillatory power at 1–49 Hz using wavelets of 7-cycle length. Frequency resolution was set to 1 Hz and time resolution to 10 ms. Within each epoch, we then averaged 5-time windows of 10 ms, producing 8 time-segments. Next, we calculated the spectral power difference before (PRE) and (POST) after tACS. PRE and POST contained ~ 80 epochs for task-based data and ~170 epochs for RSEO and RSEC data.

To calculate POST–PRE power difference (ΔPOST–PRE), we randomly shuffled the order of the epochs along the time axis in PRE and in POST (see illustration in Fig. 2). No other domain was shuffled. ΔPOST–PRE power for each shuffled trial pairs, each time-segment (8) and each electrode (62) was normally distributed and used as input for the classifier (*mv_classify.m*). This input was three-dimensional: All epochs per class × electrodes × time-segments, the latter specified to be the feature domain. In addition, we provided the classifier with a vector of class-labels, stating to which stimulation session belongs each of the epochs. Classification was performed at each frequency component in a 4–49-Hz range separately. This means that the classifier searched for the optimal time-segments within the specified time window (see above) for the classification process. The performance of the classifier was then evaluated for each electrode.

For classification, data were first projected into a 2-dimensional discriminative subspace. Then, an optimal linear transformation of the data was applied to maximize class separability. To counteract over-fitting, a regularization parameter λ was estimated. The procedure entailed: training, testing, and cross-validation. During training, the parameters of the model were optimized to discriminate between the three classes (lMC-tACS, rCB-tACS, and sham). During testing, the model performance was evaluated on an independent subset of the data, which was not used for training. The training and testing phases were repeated on different subsets of the data using 10-fold cross-validation, in which the data was split into 10 different parts and in each iteration of the cross-validation phase, 1 of the 10 parts was used for testing and the other 9 parts for training. The performance of the classifier was evaluated using the *F*1-score, which is the harmonic average of precision and recall.

$$\begin{eqnarray*} &&\mathrm{Precision}=\frac{tp}{tp+ fp}\,\,\,\mathrm{Recall}=\frac{tp}{tp+ fn}{F}_1\nonumber\\ &&\mathrm{Score}=2\cdot \frac{\mathrm{precision}\cdot \mathrm{recall}}{\mathrm{precision}+\mathrm{recall}} \end{eqnarray*}$$

where tp is the number of true positives, fp—the number of false positives, and fn—the number of false negatives. Importantly, for multiclass classification, *F*1-score is the most optimal score for evaluating classification accuracy. Finally, the *F*1-score was averaged across theta (θ, 4–8 Hz), alpha (α, 9–13 Hz), beta (β, 14–30 Hz), and gamma (γ, 31–49 Hz).

### Source Reconstruction and MVPA

To reconstruct the EEG signals in source space we used the linear constrained minimum variance (LCMV) beamforming approach as implemented in Fieldtrip. As a first step we created a head model, which was used to estimate the electric field measured by the EEG electrodes. Since individual MRI scans were not available, a standard MRI template was used to construct the boundary element model. To this end, we segmented a template into three tissue types: brain, skull, and scalp. Next, we estimated for each tissue type a boundary triangle mesh (brain: 3000 points, skull: 2000 points, and scalp: 1000 points). Based on this geometry, a volume conduction model was specified (using standard tissue conduction values) using a Boundary Element Method. For each grid point, we calculated a lead field matrix, which was then used to calculate the inverse spatial filter. The inverse spatial filter was calculated across all trials (PRE & POST).

Next and following the preprocessing steps described above, signals were band-pass filtered for α and θ, in accordance with the scalp-level results below (see Better classification of α and θ power to lMC-tACS is specific to stimulation electrodes FC3 and CP3). The Hilbert transform was then applied to obtain the spectral power. Source orientation was optimized by using the orientation of maximum signal power (Sekihara and Nagarajan 2008), resulting in a reconstructed signal in the α and θ frequency bands for each grid point. Classification was performed identically to the electrode-space analysis above, only that instead of electrodes, the performance of the classifier was evaluated for each grid point. In order to prevent spurious classification results due to spatial leakage (Gohel et al. 2018), we used a parcellation procedure based on an automated Talairach atlas (Lancaster et al. 1997) as well as the AAL (automated anatomical atlas) (Tzourio-Mazoyer et al. 2002) to create regions of interest (ROIs). *F*1-scores were averaged across grid points belonging to each ROI. Based on the scalp-level results, we specified five ROIs on the left hemisphere: M1 defined as Brodmann area (BA) 4, somatosensory cortex (S1), premotor cortex (PMC, BA6), superior parietal lobule (SPL), and inferior parietal lobule (IPL; c.f., Fig. 6*A*).

### Statistical Analyses of Classification Accuracies

Subject-specific classification results were analyzed at the group level. To test for *F*1-score differences across classes (lMC-tACS, rCB-tACS, and sham) in the electrode-space signals, we used a nonparametric cluster-based Monte Carlo permutation testing with 1000 randomizations as implemented in the Fieldtrip toolbox. This analysis resulted in a *F*-value for the comparison across all classes in each electrode and each frequency band. For the source-space data, we tested for *F*1-score differences across classes using the nonparametric Kruskal–Wallis test in each of the five ROIs (M1, S1, PMC, SPL, and IPL). *P*-level threshold was false discovery rate (FDR) corrected across the five ROIs. All post-hoc tests of significant effects were performed using the nonparametric Wilcoxon signed-rank test.

### Phase-Coupling Analysis

To examine the relationship between aftereffects in θ and α frequency bands, we measured phase-coupling between θ and α oscillations using the phase-locking value (Lachaux et al. 1999) defined as follows:

$$ {PLV}_{\theta, \alpha }=\frac{1}{N}\left|\sum\ {\rm exp}\ (i[\varnothing_{\theta} (t)-\varnothing_{\alpha} (t)])\right|$$

*N* is the number of trials and $\varnothing (t)$ is the phase of the theta or alpha oscillation at each time point. POST–PRE phase-locking values (PLV) in each stimulation protocol were compared using the nonparametric Kruskal–Wallis test.

We then tested for PLV differences in POST–PRE tACS, averaged across trials, across the stimulation protocols (lMC-tACS, rCB-tACS, and sham) using Kruskal–Wallis test in electrodes FC3, C3, and CP3. *P*-level threshold was FDR-corrected across the three electrodes.

## Results

### Better Classification of α and θ Power to lMC-tACS is Specific to Stimulation Electrodes FC3 and CP3

#### Task-based analysis

**Figure 3:**
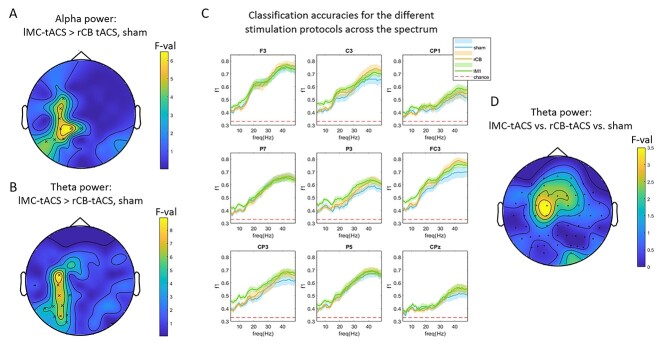
Task classification results. (*A*,*B*) Topographic plots of F-values showing differences between classification accuracies of POST–PRE, single-trial, alpha (*A*) and theta (*B*) power across stimulation protocols. (*C*) Spectral representation of classification accuracies for each stimulation protocol in electrodes of the cluster shown in (*A*). Shaded areas represent the standard error of the mean across subjects. (*D*) Topographic plot for *F*-values showing theta POST–PRE power differences (uncorrected).

First, we focused our analysis on the ΔPOST–PRE power of task-based signals. We compared classification accuracies (*F*1-score) with each stimulation protocol (lMC-tACS, rCB-tACS, and sham) using a one-way repeated measures analysis of variance (rmANOVA) and whole-brain cluster-based Monte-Carlo permutation analysis, in each frequency band. We hypothesized that successful classification of spectral α power to lMC-tACS would be significantly larger in electrodes at and adjacent to the stimulation electrodes FC3 and CP3, when compared with both rCB-tACS and sham. Note that a similar comparison for rCB-tACS against lMC-tACS and sham was not possible as there were no electrodes placed to record cerebellar oscillations (Andersen et al. 2020). For α, we found a marginal effect in left central cluster (*P* = 0.047) with maximal differences in CP1 (*F*_2,23_ = 6.5) but also CP3 (*F*_2,23_ = 5.7) and FC3 (*F*_2,23_ = 5.1; see Fig. 3*A*). For θ, a similar cluster was observed (cluster *P* = 0.016) with maximal effects at FC3 (*F*_2,23_ = 8.9) and CP3 (*F*_2,23_ = 7.4; Fig. 3*B*). No significant clusters were observed for β or γ. In Figure 3*C*, we plot the mean *F*1-score for each frequency component in each of the electrodes of the cluster identified for α (Fig. 3*A*).

Post-hoc tests revealed that the significant difference in α between the three stimulation protocols stems from larger classification accuracy for lMC-tACS compared with rCB-tACS in most electrodes of this cluster (Fig. 3*A*,*C*, *Z* > 2.1, *P* < 0.05, FDR corrected). Classification accuracy comparing lMC-tACS to rCB-tACS in θ showed significant differences in electrodes C3, CP1, P7, P3, O1, FC3, CP3, P5, PO5, PO3, and PO7 (all *Z* > 2.1, *P* < 0.05, FDR corrected). Comparing classification accuracy in lMC-tACS with sham, significant difference in θ were found for FC3 only (*Z* = 2.9, *P* = 0.003, FDR corrected). A similar difference in CP3 was found on trend (*Z* = 2.4, *P* = 0.016, no correction). For α, a similar trend (*P* < 0.05, no correction) was found in electrodes FC3 and CP3 (*Z* = 2.7, *P* = 0.008; *Z* = 2.3, *P* = 0.02).

*In sum, these results demonstrate that short segments of* α *and* θ *oscillations during task-performance following 10*-*Hz lMC-tACS could be accurately distinguished from 10*-*Hz tACS of another node in the motor network as well as from sham-stimulation, in a focal manner.*

#### Resting-State Analysis

**Figure 4:**
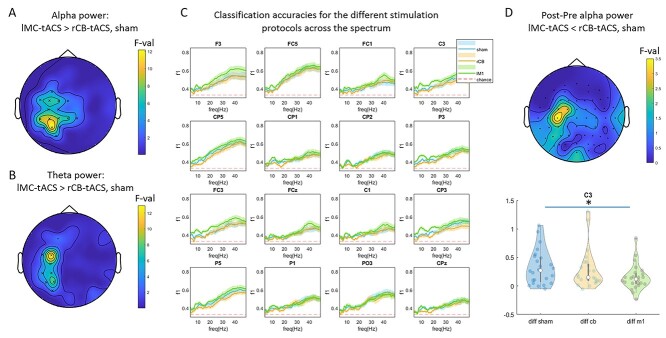
Resting-state classification results. (*A*,*B*) topographic plots of F-values showing differences between classification accuracies of POST–PRE, single-trial, alpha (*A*) and beta (*B*) power across stimulation protocols. (*C***)** Spectral representation of classification accuracy for each stimulation protocol in electrodes of the cluster shown in (*A*). Shaded areas represents the standard error of the mean across subjects. (*D*) Topographic plot for alpha POST–PRE power differences. In electrode C3 POST–PRE power was lowest for lMC-tACS compared with both rCB-tACS and sham (violin plot).

Next, we performed a similar analysis in resting-state eyes-open (RSEO) as well as resting-state eyes-closed (RSEC) of ΔPOST–PRE power. In accordance with the task-based signals, a significant left-central cluster was evident in α (*P* = 0.016) for RSEO. The cluster for RSEO had maximal effects in electrode P3 (*F*_2,23_ = 12.2, see topoplot in Fig. 4*A*), adjacent to the stimulation electrode CP3. A strong effect was found also in FC3 (*F*_2,23_ = 6.5). For θ, a similar cluster was also found (Fig. 4*B*, cluster significance: *P* = 0.04) with a maximal effect in FC3 (*F*_2,23_ = 12.9). Figure 4*C* shows the mean *F*1-score for each frequency component. No clusters were evident for RSEC.

Post-hoc tests on classification effects in α revealed that the significant difference between the three classes stemmed from larger classification accuracy for lMC-tACS compared with rCB-tACS in the entire cluster (Fig. 4*A*,*C*, all *Z* > 2.2, *P* < 0.05, FDR corrected). For θ, classification accuracy was larger for lMC-tACS compared with rCB-tACS as well in the entire cluster (Fig. 4*B*, all *Z* > 2.2, *P* < 0.05, FDR corrected). Compared with sham, accuracy for lMC-tACS in α was significantly larger in electrodes CP3 (*Z* = 3.1, *P* = 0.002) and P3 (*Z* = 3.4, *P* < 0.001). For θ, accuracy for lMC-tACS tended to be larger compared with sham in electrodes P3 (*Z* = 2.2, *P* = 0.028) and FC3 (*Z* = 2.4, *P* = 0.018).

*These results suggest that short segments of* α *and* θ *oscillations during eyes-open resting-state following 10*-*Hz lMC-tACS could be accurately distinguished from other stimulation protocols in a focal manner.*

### No Associations between Classification Accuracies and Motor Performance

Next, we asked whether better classification of θ and α power post-stimulation is driven by behavioral changes during task-performance due to tACS. In the SRTT, subjects perform stimulus–response matching and their performance is measured by reaction times. We therefore correlated the classification accuracies in electrodes FC3 and CP3 in each stimulation protocol with averaged reaction-time differences, across all trials, between PRE and POST blocks, of each stimulation protocol. We found no evidence to support an association between task performance and classification accuracies at θ and α in electrodes FC3 and CP3 in any of the stimulation protocols (*P* > 0.05).

### The Effect of tACS on θ/α Power Differences

Next, we averaged oscillatory power across all trials during task performance in PRE and POST tACS blocks, in order to determine the direction of power changes following tACS. Then, we assessed ΔPOST–PRE power between the stimulation protocols using whole-brain Monte-Carlo permutation analysis. There were no significant differences between the stimulation protocols on a corrected level (cluster-based, *P* < 0.05). However, electrodes FC1 (*F*_2,23_ = 3.7, *P* = 0.02) and C1 (*F*_2,23_ = 3.2, *P* = 0.037) showed a tendency for a significant difference across stimulation protocols in θ (Fig. 3*D*). Post-hoc Wilcoxon signed rank tests showed no significant differences. There were no differences evident for α.

A similar analysis of RSEO signals revealed no significant differences between stimulation protocols on a corrected level (cluster-based *P* < 0.05), but electrode C3 showed evidence for differences in α power (*F*_2,23_ = 3.7, *P* = 0.02, Fig. 4*D*). Indeed, post-hoc Wilcoxon signed rank tests showed that ΔPOST–PRE power under lMC-tACS were smaller compared with both rCB-tACS (*Z* = 2.3, *P* = 0.02) and sham (*Z* = 2.0, *P* = 0.045). No such effects were observed for θ. There was no correlation between ΔPOST–PRE power and *F*1-scores in any of the classes.

*These results show that 1) traditional analysis of power effects averaged across trials are far less sensitive compared*  *with MVPA. 2) classification differences between lMC-tACS and rCB-tACS/sham result from a decrease in resting-state* α *power post-stimulation. A similar conclusion for task-based EEG could not be derived.*

### Association between Aftereffects of tACS on θ and α Frequency Bands

**Figure 5:**
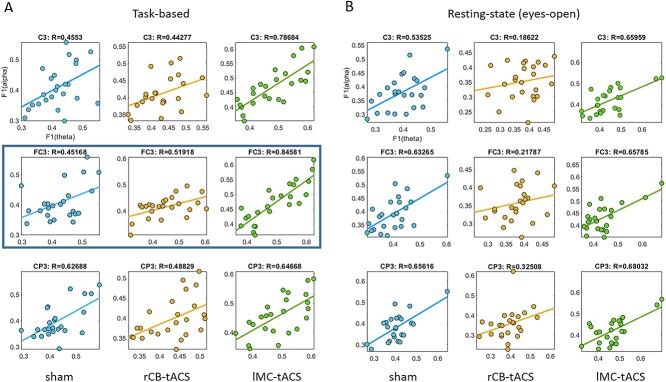
Correlation between theta and alpha classification results. *F*1-score for theta and alpha frequency bands was strongly correlated for lMC-tACS in both task-based (*A*) and resting-state signals (*B*). In electrode FC3 and task-based signals (marked with a frame), this correlation was larger compared with both rCB-tACS and sham.

We further explored whether classification accuracy in electrodes FC3, C3, and CP3 in α was associated with classification accuracy in θ, which would raise the possibility that aftereffects in θ were driven by the stimulation frequency at 10 Hz. Indeed, in the task-based signals, *F*1-scores at θ were correlated with *F*1-scores at α for lMC-tACS in FC3, C3, and CP3 (*r* = 0.85, 0.79, 0.65, all *P* < 0.001, Fig. 5*A*). Correlations were also evident for rCB-tACS (*P* = 0.009, 0.04, 0.016, resp., Fig. 5*A*) as well as sham (*P* = 0.03, 0.03, 0.001, resp., Fig. 5*A*).

Using Fisher *Z*-transformation, we found that correlation between *F*1-scores in θ/α in electrode FC3 was significantly stronger in lMC-tACS compared with rCB-tACS (*P* = 0.03) and sham (*P* = 0.01). Similarly, a correlation analysis between classification accuracies in α and θ for RSEO signals, showed a strong association in electrodes FC3, C3, and CP3 for lMC-tACS (*r* = 0.66, 0.66, 0.68, *P* < 0.001, see Fig. 5*A*). Here however, the correlation coefficients did not differ between lMC-tACS and rCB-tACS/sham in any of the electrodes (*P* > 0.06, uncorrected). *These results suggest that better classification accuracy in lMC-tACS at the* θ *band was associated with and perhaps driven by better classification accuracy in the* α *band for lMC-tACS, specifically for task-based signals.*

To tap into possible mechanisms of these associations, we further explored θ/α phase-coupling in electrodes FC3, C3, and CP3, averaged across all trials in PRE and POST tACS blocks. We found no differences in phase-coupling between stimulation protocols for task-based signals (all *P* > 0.4). For RSEO signals, significant differences between the stimulation protocols were found in FC3 (*P* = 0.01, data not shown). Post-hoc Wilcoxon signed-rank tests showed that this effect stemmed from increased θ/α phase-coupling in rCB-tACS compared with sham (*Z* = 2.7, *P* = 0.006) and a tendency for larger phase-coupling in lMC-tACS compared with sham (*Z* = 1.7, *P* = 0.08). *These results suggest that the stronger associations between classification accuracies in* θ *and* α *under lMC-tACS are likely not driven by phase-coupling mechanisms.*

### Localization of Classification Effects in Source-Space

**Figure 6:**
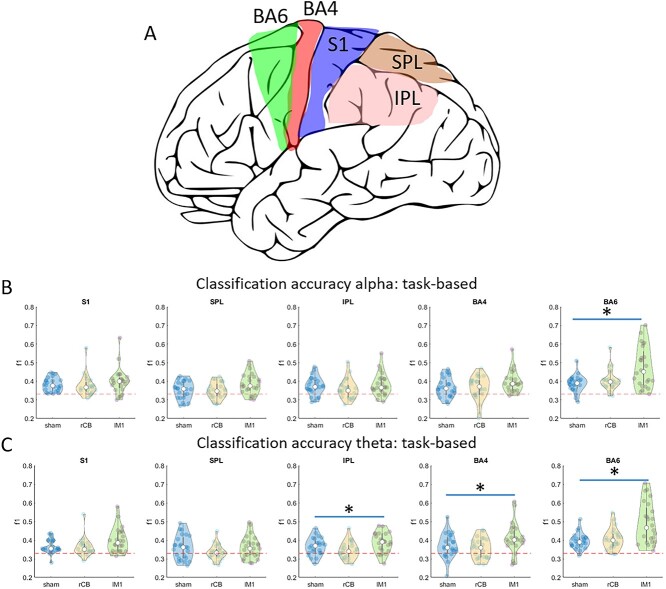
Classification of task-based signals in source space. (*A*) illustration of regions-of-interest included in the source-space analysis. (*B*,*C***)** classification accuracies averaged across all voxels in somatosensory cortex (S1), primary motor cortex (BA4), premotor cortex (BA6), SPL and IPL in alpha (*B*) and theta (*C*) frequency bands.

To further study the spatial topography of better classification of ΔPOST–PRE in α and θ power to lMC-tACS compared with rCB-tACS and sham, we used LCMV beamforming to reconstruct classification accuracies in source-space. We focused our analysis on the following left hemispheric ROIs: M1, S1, PMC, SPL, and IPL, based on the scalp-level results (Fig. 6*A*). As classification accuracies at source-level were lower compared with scalp-level, we first evaluated in each ROI whether classification was better compared with chance level (approximated at 34% across subjects and classes, FDR corrected for multiple comparisons across all ROIs).

**Figure 7:**
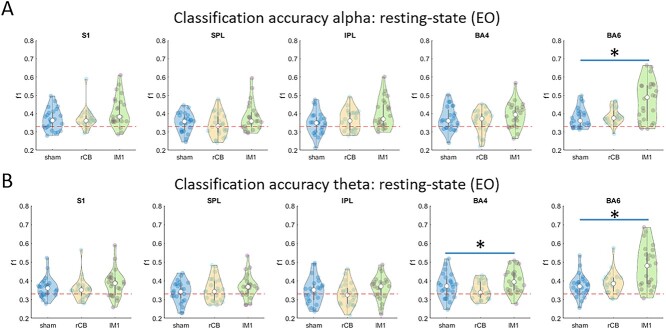
Classification resting-state signals in source space in alpha (*A*) and theta (*B*) frequency bands.

#### Task-Based Signals

Across all classes, classification accuracies of reconstructed α power in ROIs: M1, S1, PMC, and SPL were significantly better than chance level. For reconstructed θ power, classification accuracies in S1 and PMC were better than chance level across all classes.

We then compared classification accuracies of α and θ power across the three stimulation protocols in each ROI. For α, we found a significant effect in PMC (χ^2^ = 10.3, *P* = 0.006, Fig. 6*B*). Post-hoc tests revealed that this effect stemmed from a larger classification accuracy in PMC comparing lMC tACS with sham (*Z* = 3.0, *P* = 0.003) as well as to rCB tACS (*Z* = 2.3, *P* = 0.02). For θ (Fig. 6*C*), a significant effect was found in IPL (χ^2^ = 8.3, *P* = 0.016), M1 (χ^2^ = 8.5, *P* = 0.014) and PMC (χ^2^ = 11.5, *P* = 0.003). Post-hoc tests revealed that these effects stemmed from a significant difference in classification accuracy in PMC comparing lMC-tACS with sham (*Z* = 3.1, *P* = 0.002) and in M1, comparing lMC-tACS with rCB-tACS (*Z* = 2.9, *P* = 0.004).

#### Resting State

Classification accuracy of reconstructed α power in RSEO was better than chance level across all classes in ROIs: S1, M1, and PMC. Significant difference in classification accuracies across classes was observed only in PMC (χ^2^ = 10.7, *P* = 0.005, Fig. 7*A*). Post-hoc tests revealed that these effects stemmed from larger classification accuracy comparing lMC-tACS with sham (*Z* = 2.8, *P* = 0.005) and with rCB-tACS (*Z* = 2.3, *P* = 0.02).

Classification accuracy of reconstructed θ power in RSEO was better than chance level across all classes, only in PMC. Significant differences in classification accuracies across classes were observed in M1 (χ^2^ = 8.3, *P* = 0.02) and PMC (χ^2^ = 13.3, *P* = 0.001, Fig. 7*B*). Post-hoc tests in PMC revealed larger classification accuracy in lMC-tACS compared with sham (*Z* = 3.2, *P* = 0.002) and to rCB-tACS (*Z* = 2.5, *P* = 0.01).

*These results suggest that better classification of short segments of* θ *and* α *oscillations to lMC-tACS, observed in wide-spread left centro-parietal clusters on the scalp-level, could be reconstructed to a source residing in left PMC.*

## Discussion

In this study, we examined the “offline” aftereffect of 10-Hz tACS at key nodes of the motor network on oscillatory power following stimulation. To this end, we extracted short EEG segments during motor task performance and at rest, and compared the ability of a machine-learning algorithm (i.e., LDA) to correctly classify these segments to one of three stimulation protocols: lMC-tACS, rCB-tACS, or sham. This novel approach allowed to statistically assess in a data-driven manner whether these short EEG signals contain neuroplastic signatures of tACS at the stimulation frequency.

Our results show that 10 Hz tACS aftereffects are: 1) Location-specific. Better classification of oscillatory power to lMC-tACS compared with rCB-tACS/sham was maximal in electrodes FC3 and CP3, which were used for lMC-tACS stimulation. 2) Broad-band. Effects were found in both α (9–13 Hz) and θ (4–8 Hz) frequency bands but were probably driven by the α oscillation. 3) Generalizable. Effects were evident during motor task performance as well as at eyes-open rest condition. And finally, 4) originating from the PMC evident by source-localization analysis.

The broad-band effect, spanning both θ and α frequency bands, agrees with previous reports indicating that oscillatory power following tACS was modulated in frequency bands beyond the actual tACS frequency. For example, 5-Hz tACS over the frontal cortex during non-rapid eye movement sleep, decreased overall oscillatory spectral power in low (0.5–4 Hz) frequencies as well in the α band at the stimulation electrodes (Marshall et al. 2011). Others found increased delta power following iAF-tACS (Neuling et al. 2012). We found that classification accuracy in θ and α were correlated in FC3 and more strongly for lMC-tACS compared with rCB-tACS/sham, suggesting that these effects were strongly associated and perhaps driven by the 10-Hz stimulation. According to Veniero et al. (2015), such associations in tACS aftereffects may be rooted in cross-frequency coupling mechanisms. We found, however, no evidence to support this assertion in our data, suggesting that that phase coupling is an unlikely mechanism to explain multi-band aftereffects of 10-Hz tACS to the motor cortex. Another possible mechanism underlying this broad-band aftereffect could be the nonlinear “Arnold tongue” observed in “online” entrainment at larger stimulation intensities (Herrmann and Strüber 2017). “Arnold tongue” describes a region of stimulation amplitudes and frequency pairs that produces high synchrony between the stimulation waveform and endogenous oscillations. In a set of experiments with ferrets, Huang et al. (2021) showed that tACS entrains alpha oscillations following the “Arnold tongue” principle in posterior parietal cortex neurons. Whether and how long this effect persists after stimulation terminates still need to be determined. In a computational simulation of tACS aftereffects on a neural network model, incorporating spike-timing dependent plasticity rules, long-term potentiation (LTP) of synaptic strength was induced when stimulation was at the frequency of the intrinsic oscillation. LTP was accompanied by an increase of EEG spectral power (Zaehle et al. 2010). In contrast, synaptic weakening occurred when stimulation was performed at a frequency slightly faster than the spontaneous frequency (Vossen et al. 2015), conceivably resulting in decreased power post-stimulation. Here, we observed α power decrease (during resting-state, averaged across trials) in electrode C3 under lMC-tACS compared with rCB-tACS/sham. Thus, intrinsic frequencies below 10 Hz may have led to synaptic weakening and location-specific aftereffects at both α and θ frequencies (Vossen et al. 2015). However, as previous report (Gundlach et al. 2017) found decreased power using iAF-tACS, the significance of small deviations between intrinsic and external frequencies remains open.

Of note, we found better classification accuracy with higher frequencies, regardless of the specific stimulation protocol or electrode examined. This could be a result of increased performance by LDA when transient changes in the signal take place at higher frequencies. Note that there were no differences between the stimulation protocols in terms of performance at higher frequencies.

Interestingly, although we find strong evidence to support better classification of oscillatory θ/α power comparing lMC-tACS with rCB tACS/sham in both motor task and resting-state signals, only resting-state (eyes-open) α power yielded a significant location-specific decrease when data were averaged across trials. This dissociation testifies for the sensitivity of the multivariate approach in detecting subtle changes that are perhaps overlooked by traditional statistical methods with a multitude of temporal and spatial parameters requiring strict statistical correction procedures. Indeed, a recent study has examined aftereffects of 20 min high-density 10 and 20-Hz tACS to bilateral sensorimotor areas and found no evidence of modulation of averaged alpha or beta power post-stimulation (Lafleur et al. 2021). Although this lack of effect might be due to the experimental design (no task was used during stimulation to activate the motor network), it strengthens the argument that traditional epoch-averaging is not sensitive enough for detecting the subtle aftereffects of motor cortex tACS.

Importantly, electrode-space MVPA revealed better classification accuracy for lMC-tACS, not only at and around the stimulation electrodes FC3 and CP3 but also at left parietal electrodes. This result could be indicative for the lack of focal effects by tACS and therefore it was essential to investigate whether a specific region showed neuroplastic effects following stimulation using source reconstruction methods. To this end, we analyzed differences in classification accuracies on reconstructed signals from left M1, S1, PMC, SPL, and IPL. We found that classification accuracies in left PMC were significantly stronger comparing lMC-tACS with rCB-tACS/sham for both α and θ in both task and rest signals. This result testifies for more robust aftereffects in PMC, and less at M1, as the computational model of current distribution (Supplementary Material Section 1, Fig. 1*C*) suggested. A possible explanation to this finding is that while both M1 and PMC are stimulated by tACS, neural activity in PMC is less heterogeneous across subjects, producing a more reliable aftereffect. Support for this notion is found in a focal tDCS study targeting both premotor and M1. Results show larger MEPs when targeting PMC compared with M1, leading the authors to suggest more reliable changes in cortical excitability are produced by PMC tDCS (Lefebvre et al. 2019).

## Limitations

A few limitations of our study need to be mentioned. First, the cerebellum stimulation site was included as an active control for better localization of motor cortex tACS effects. However, as we did not have EEG recordings from the cerebellum, we were not able to investigate aftereffects of cerebellar tACS compared with M1 tACS and sham. Future tACS-EEG studies exploring the motor network should consider including recordings of cerebellar oscillations (Andersen et al. 2020) to be able to answer whether cerebellar tACS induces localized tACS aftereffects. Second, the aim of the study was to elucidate tACS aftereffects on the motor system and to this end we used a relatively short time window after tACS. However, these aftereffects may last as long as 70 min (Kasten et al. 2016). Future studies could address the question of how long tACS aftereffects persist by examining aftereffects using MVPA in later time windows following tACS.

## Conclusion

Using a novel machine-learning approach, we found that short α/θ oscillations recorded after stimulation, could be better classified to lMC-tACS compared with rCB-tACS/sham and thus serve as a signature for location-specific neuroplasticity following 10 Hz tACS, independent of the state (both at task and rest signals). These results elucidate the effects of tACS over the motor cortex and thus have important consequences for therapeutic interventions using tACS.

## Supplementary Material

Supplementary_materials_tgab067
