# Supplementary material for: Classification of EEG Signals Reveals a Focal Aftereffect of 10 Hz Motor Cortex Transcranial Alternating Current Stimulation
Source: Cereb Cortex Commun. 2022 Jan 7;3(1):tgab067. doi: 10.1093/texcom/tgab067 (PMC8790173; doi:10.1093/texcom/tgab067)
Supplement: Supplementary_materials_tgab067 [file supplementary_materials_tgab067.docx]

# Supplementary materials

# Classification of EEG signals reveals a focal aftereffect of 10Hz motor cortex transcranial alternating current stimulation

Elinor Tzvi, Jalal Alizadeh, Christine Schubert, Joseph Classen

Department of Neurology, Leipzig University

**B**

**A**

500ms


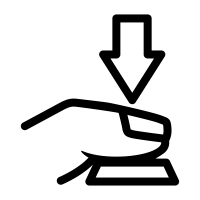


Time


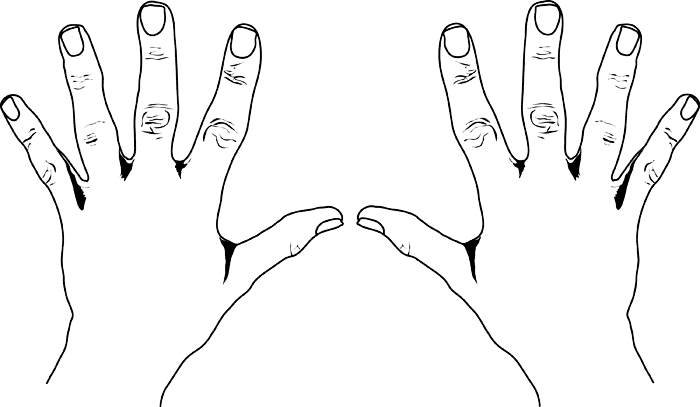


**C**

**PRE-tACS**

**tACS**

**POST-tACS**

RND

RND

SEQ

120x4

SEQ

…

SEQ

…

120x3

120

80

SEQ

40

120

40

40

RND

80

80

SEQ

120

RND

80

SMP

RND

RND

RND

***Supp. Figure 1.*** Serial reaction time task. In each trial, 4 black squares were presented. At stimulus onset, one of the squares turned blue and subjects were instructed to press the button corresponding to the blue square with the respective finger. **B** Task timeline.

1. **Computational modelling of MC and cerebellar-stimulation locations**

We used the SimNIBS software package (http://simnibs.org/), version 2.1 (Saturnino et al., 2019), to simulate the optimal electrode montage for focal left motor cortex (lMC) and right cerebellar (rCB) electric stimulation. The head model, provided by the software package, was created using finite element modeling on T1- and T2-weighted MRI images of an exemplary subject, resulting in a high-resolution tetrahedral head mesh model containing 6 tissue types (grey matter (GM), white matter (WM), cerebrospinal fluid (CSF), skull, skin and eye balls). We set the following standard conductivity values for the 6 tissue types: WM: 0.126 S/m, GM: 0.275 S/m, CSF: 1.654 S/m, skull: 0.01 S/m, eye balls: 0.500 S/m as well as the following conductivity values for the electrode rubber = 29.4 S/m and the electrode gel = 1.0 S/m. All tissues were treated as isotropic. The electrical field E was determined by taking the numerical gradient of the electric potential. For both montages, we used ring-shaped electrodes with 48mm outer diameter, 24mm inner diameter and 3mm thickness. The size and geometry of the electrodes were incorporated into the forward model. The total current injected was 1mA. For lMC-tACS montage, electrodes were placed at EEG locations FC3 and CP3. For rCB-tACS montage, one electrode was placed 1cm below and 3cm right to the inion and the other over right mandibula.

1. **Classification comparison between LDA and SVM**

To evaluate the performance of linear discriminant analysis (LDA) in correctly classifying EEG segments following tACS, we compared classification performance with support vector machines (SVM), a method commonly employed for EEG data (e.g., Obleser and Weisz, 2012). To this end, we compared two classes only: M1 tACS and sham, since SVM implementation in MVPA-light (and its extension [LIBSVM](https://www.csie.ntu.edu.tw/~cjlin/libsvm/)) does not allow for 3-class comparison as for LDA. All parameters for LDA were kept the same as in the main text. For SVM, we specified a linear kernel. Classification accuracy, representing the fraction of correctly predicted class labels, was estimated across both MC tACS and sham. We used paired-sampled t-test to directly compare for each electrode and each frequency component, whether LDA or SVM produced a higher classification accuracy. The results are presented in supp. Fig. 2. We found that SVM performs significantly better for electrodes around MC tACS stimulation location (FC3, CP3) in the theta frequency range (4-8Hz) but the improvement is not large (2-3%). For higher frequencies, LDA outperforms SVM, again by 2-3% (electrode CP3, see supp. Fig. 2 below). Note that we did not find differences between the classes using multi-class LDA in higher frequencies but observed a general increase in performance when compared to lower frequencies. Nonetheless these differences may testify for tACS aftereffects on oscillations beyond the stimulation frequency which were not detected when testing across 3-classes (in the main text).


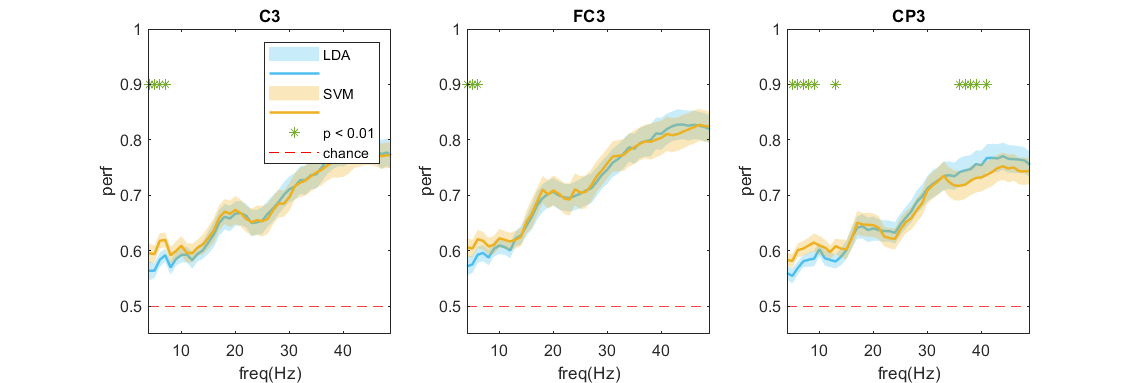

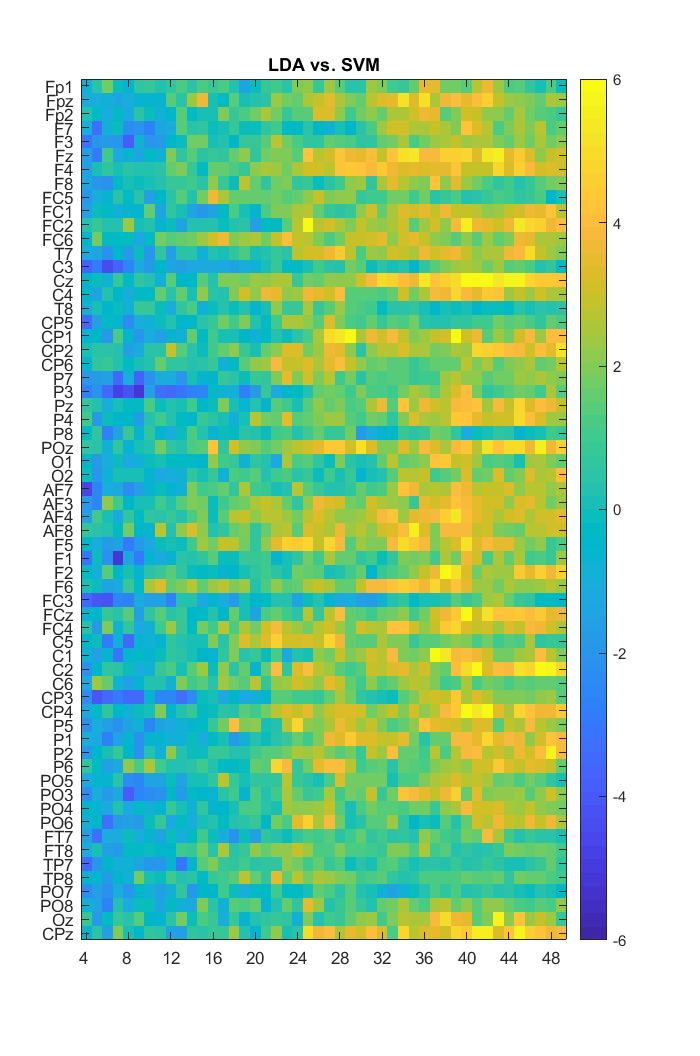


Supplementary Figure 2. Comparison of classification accuracies for LDA and SVM across MC tACS and sham. Upper panel: performance of both algorithms at different frequencies for the stimulation electrodes FC3 and CP3 as well as C3. Shaded areas represent the standard error of the mean across subjects. Green asterisks mark the frequencies in which the performance difference was significant (p<0.01, uncorrected). Bottom panel: overview of performance difference across all electrodes and frequencies. Color bar is t-value for the comparison between the methods. Blue: SVM>LDA, yellow: LDA>SVM.

1. **General tACS effects**

None of the subjects reported any adverse effects during or after stimulation. We asked subjects whether they experienced phosphenes, which could be a sign of visual cortex stimulation (Kar and Krekelberg, 2012). Seven out of 25 subjects reported phosphenes during either lMC-tACS (N = 2) or rCB-tACS (N = 5). Three of the seven claimed to see phosphenes during sham. There were no reports on pain or dizziness due to stimulation. During real tACS when asked whether the session was sham, subjects correctly answered “no” in 48% of all real tACS sessions. During sham, 19 out of 25 subjects answered correctly that the session was sham, which might indicate that they were aware of the intervention. However, it seems that the formulation of the question (“was this session sham?”) has led to this large number of subjects correctly identifying the sham session. For example, five out of 25 subjects answered “yes” in all sessions, and 13 out of 25 subjects answered “yes” in two out of three sessions.

1. **No associations between classification accuracies and motor performance**

To explore whether better classification of theta and alpha power post-stimulation may relate to performance changes due to tACS, we correlated F1-values for electrode FC3 and CP3 with reaction-time differences between PRE and POST blocks. We found no evidence to support an effect of motor performance on classification accuracies of theta and alpha power in electrode FC3 and CP3 (data not shown).

## References

Kar, K., Krekelberg, B., 2012. Transcranial electrical stimulation over visual cortex evokes phosphenes with a retinal origin. J. Neurophysiol. https://doi.org/10.1152/jn.00505.2012

Obleser, J., Weisz, N., 2012. Suppressed Alpha Oscillations Predict Intelligibility of Speech and its Acoustic Details. Cereb. Cortex 22, 2466–2477. https://doi.org/10.1093/CERCOR/BHR325

Saturnino, G.B., Puonti, O., Nielsen, J.D., Antonenko, D., Madsen, K.H., Thielscher, A., 2019. SimNIBS 2.1: A Comprehensive Pipeline for Individualized Electric Field Modelling for Transcranial Brain Stimulation, in: Brain and Human Body Modeling. https://doi.org/10.1007/978-3-030-21293-3_1
